# Deploying a quantum annealing processor to detect tree cover in aerial imagery of California

**DOI:** 10.1371/journal.pone.0172505

**Published:** 2017-02-27

**Authors:** Edward Boyda, Saikat Basu, Sangram Ganguly, Andrew Michaelis, Supratik Mukhopadhyay, Ramakrishna R. Nemani

**Affiliations:** 1 Department of Physics and Astronomy, Saint Mary’s College of California, Moraga, CA, United States of America; 2 Bay Area Environmental Research Institute, Moffett Field, CA, United States of America; 3 Department of Computer Science, Louisiana State University, Baton Rouge, LA, United States of America; 4 Earth Science Division, NASA Ames Research Center, Moffett Field, CA, United States of America; 5 University Corporation at CSU Monterey Bay, Seaside, CA, United States of America; 6 NASA Advanced Supercomputing Division, NASA Ames Research Center, Moffett Field, CA, United States of America; Kerala Forest Research Institute, INDIA

## Abstract

Quantum annealing is an experimental and potentially breakthrough computational technology for handling hard optimization problems, including problems of computer vision. We present a case study in training a production-scale classifier of tree cover in remote sensing imagery, using early-generation quantum annealing hardware built by D-wave Systems, Inc. Beginning within a known boosting framework, we train decision stumps on texture features and vegetation indices extracted from four-band, one-meter-resolution aerial imagery from the state of California. We then impose a regulated quadratic training objective to select an optimal voting subset from among these stumps. The votes of the subset define the classifier. For optimization, the logical variables in the objective function map to quantum bits in the hardware device, while quadratic couplings encode as the strength of physical interactions between the quantum bits. Hardware design limits the number of couplings between these basic physical entities to five or six. To account for this limitation in mapping large problems to the hardware architecture, we propose a truncation and rescaling of the training objective through a trainable metaparameter. The boosting process on our basic 108- and 508-variable problems, thus constituted, returns classifiers that incorporate a diverse range of color- and texture-based metrics and discriminate tree cover with accuracies as high as 92% in validation and 90% on a test scene encompassing the open space preserves and dense suburban build of Mill Valley, CA.

## Introduction

The proliferation of very high resolution (VHR) aerial and satellite imagery opens the way to significant improvements in remote sensing data products. It is now possible to identify structures at better than 1-meter resolution, down from 30 meters in existing Landsat-based solutions. Objects—individual sheds, tractors, streams, islands, rocks, trees, vines, and furrows—come into focus from out of broad swaths of forest or field, allowing for detailed site-specific studies as well as more accurate delineations of land cover in the large. VHR datasets are rich in potentialities. At the same time, newly sophisticated computer algorithms are required to parse the data.

Due to high variability within classes and in atmospheric, lighting, and photo-geometric conditions, land-cover class cognition at very high resolution remains a difficult challenge. In this realm, object-oriented techniques for integrated segmentation and classification have shown great recent promise. They offer a richer semantics and more accurate classification when compared to clustering of spectral and textural primitives alone. (See, for example, [1] in the context of computer vision or [2] for a review in the context of remote sensing.) Object-oriented appraoches put significant demands on computational infrastructure. The machine learning algorithms lead to memory- and processor-intensive training (optimization) problems in which thousands of parameters must be determined, while the relevant VHR datasets themselves extend to terabytes in size. Given these pressures, it is natural to ask what sorts of breakthroughs, algorithmic or technological, may lie on the horizon.

Quantum computing (see, e.g., [3]) is one such possibility. Broadly defined, quantum computing is an effort to encode hard computational problems in the dynamics of quantum physical systems. The state space of quantum systems is exponentially large in the number of basic physical variables, and if tapped properly, can yield computational results exponentially faster than the best available classical alternatives. This advantage has been demonstrated formally for particular problems, integer factorization [4] being the example most often cited due to its role in the widely-used RSA public-key cryptography scheme. The community is actively working to characterize the scaling advantages we can expect for broader classes of problems.

Within the quantum computing paradigm, quantum annealing [5–7] is a computational metaheuristic designed to solve optimization problems. Akin to simulated annealing, quantum annealing seeks the minimum of a cost function in a complex configuration space. Physically, the cost function encodes as the system’s energy. The algorithm proceeds by preparing the system in a quantum superpostion of all possible configurations in the solution space, all equally probable, thus initiating a uniquely quantum parallel processing. The system then is evolved in time until the sought minimal energy configuration is overwhelmingly probable. In principle, in the absence of thermal noise, it can be arranged so that the minimal energy configuration will be measured on read-out with probability arbitrarily close to one. Rather than sampling, physical interactions between quantum bits drive the system to the energy minimum. As part of this process, the system has the possibility of quantum tunneling through tall, narrow barriers in the energy landscape to escape local minima in less than exponential time.

A quantum annealing processor built by D-wave Systems, Inc., with 1152 quantum bits (qubits) is now operating at NASA’s Ames Research Center. The deployment of the D-wave 2X follows earlier trials of 128-qubit and 512-qubit processors at Lockheed Martin and at Ames. Much work has gone to characterize the performance of these machines. Evidence of the persistence of quantum coherence during computation has been observed in subsystems of eight qubits [8–10]. On the other hand, the processor has handily been beaten for speed by desktop CPUs running optimized simulated annealing and/or more targeted sampling algorithms [11–13]. In late 2015, a first set of problems were crafted on which the D-wave quantum annealer runs significantly faster than classical simulated annealing [14].

Motivated to advance our remote sensing capabilities and to better understand the possibilities of quantum annealing vision algorithms, we set out to train a production-scale classifier of aerial imagery on the D-wave processor. We begin with an implementation of a boosting algorithm known as QBoost, developed specifically for the D-wave architecture. It was employed in 2009 to identify cars in photographs of street scenes, having been trained on a processor with 52 functioning qubits [15–17]. Unfortunately, QBoost, along with problems from a common general class of quadratic training objectives, does not scale well on the D-wave architecture or on any foreseeable quantum annealing processor. By truncating and rescaling couplings in the QBoost training objective, with the introduction of an additional trainable metaparameter, we are able to map problems of hundreds of variables to the D-wave chip and to build the desired classifier of tree cover in aerial imagery.

This work is an offshoot of a prototype study [18] planned eventually to deliver tree cover estimates for the continental United States via 1-meter-resolution VHR data from the National Agriculture Imagery Program (NAIP) [19]. The object-oriented platform produces pixelwise probabilistic maps for tree cover as the output of a conditional random field, which itself integrates outputs from a region-merging segmentation routine and a neural network classifier. In the prototype, tree cover maps were generated for 11,095 input NAIP tiles covering the state of California, with correct detection rates of 85% in regions of fragmented forest and 70% for urban areas. We have formulated the boosted classifier so that it can work in concert with or stand in for the neural network in the larger object-oriented platform. Although this remains work in progress, we are aiming at a viable scientific application of D-wave output in the near term. Our contributions include the demonstration of tree cover classification, along with a detailed analysis of training on our remote sensing data and a shortcut solution to embed this class of problems into the D-wave architecture. Inter alia, we discovered some simple, classically fast-to-train quadratic decision stumps on derived image features that themselves produce surprisingly good classification of tree cover in California. For point of reference, antecedent case studies of potential D-wave applications include [20–27], while [28] presents a broad collection of potential applications of interest to NASA.

The paper will proceed as follows. We first review the structure of problems amenable to solutions on the D-wave quantum annealing processor. Mathematically, they are quadratic unconstrained binary optimization (QUBO) problems, and in physics, they are generalized Ising models of a spin glass. We discuss QBoost in this context, the problem of embedding into the D-wave architecture, and our proposed modifications to QBoost. We present the details of our implementation on the NAIP dataset, laying out the two problems, one on 108 qubits, another on 508 qubits, which are the focus of this study, along with our results. We conclude with a discussion of challenges and possible improvements to this framework.

## Quantum annealing on the D-wave processor

In quantum mechanics the energy function is known as the Hamiltonian, denoted *H*. It encodes all dynamics of a system and will vary with time *t* along with ambient conditions. The basic process of quantum annealing is to interpolate physically between an initial Hamilitonian *H*_0_, with an easy-to-implement minimal energy configuration (or ground state), and a problem Hamiltonian *H*_*P*_ whose minimal configuration is sought. For instance, for a linear interpolation schedule and computation time *τ*,

$$\begin{array}{c} H\left(t\right)=\left(1-\frac{t}{\tau }\right)H_{0}+\frac{t}{\tau }H_{P}. \end{array}$$ (1)

The interpolation is effected physically on the D-wave chip by adjusting currents that flow to individual qubits, each of which is a tiny superconducting circuit. The system begins in the ground state of *H*_0_ and ends, ideally, in the ground state of *H*_*P*_. For perfectly isolated quantum systems, the ground state of *H*_*P*_ can be attained for sufficiently large *τ* with probability arbitrarily close to one. In practice, due to thermal noise and loss of quantum coherence, optimal compute times in the D-wave device are actually less than its currently minimal allowable time, *τ* = 20*μ*s. [11] In this context, it should be noted that the parameter *τ* captures only the actual annealing time and does not include times for cooling, initialization, and read out of the device.

Because of the facility of physical control attainable with binary qubits and pairwise interactions between them, the problem Hamiltonian takes the form:

$$\begin{array}{c} H_{P}=-\sum_{i\in V}h_{i}s_{i}-\sum_{\{i,j\}\in E}J_{ij}s_{i}s_{j}. \end{array}$$ (2)

In physics this Hamiltonian was first studied as the Ising model of a magnet. The binary variables *s*_*i*_ ∈ {−1, +1} are thus called spins, fixed in a lattice graph G with vertices and edges (V,E). The programmable elements are the local magnetic fields, *h*_*i*_, and the couplings between spins, *J*_*ij*_. Both are in principle continuum real variables but are in practice limited to a discretum by noise in the device. The optimization seeks the minimum of *H*_*P*_ over all configurations of the spins {*s*_*i*_}.

The intuition for the optimization is as follows: The negative sign in the first term indicates that the energy is lower when a spin *s*_*i*_ aligns with (has the same sign as) the magnetic field *h*_*i*_ at lattice site *i*; this imperative competes with the demand that *s*_*i*_ align or anti-align with neighboring spins *s*_*j*_, according to the sign of the coupling *J*_*ij*_. If *J*_*ij*_ > 0, the coupling between spins is *ferromagnetic*, driving them to align. If *J*_*ij*_ < 0, the coupling is *antiferromagnetic*, driving them to anti-align. The problem of minimizing the Ising energy function with antiferromagnetic couplings is known to be NP-hard, meaning that the computational effort required for the hardest instances scales exponentially with problem size for all known classical algorithms [29, 30].

**Fig 1:**
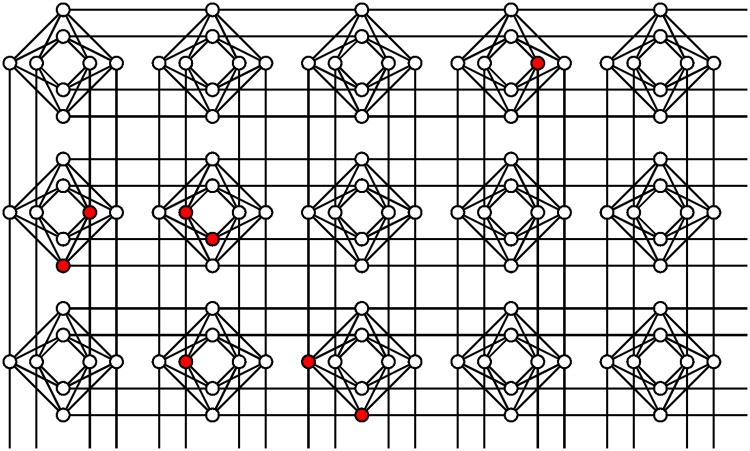
Chimera structure of qubit connectivity on the D-wave 2X processor. The full 1152-qubit graph extends to a 12x12 lattice of groups of eight qubits. Within the illustrated subset, currently inoperable qubits are marked in red.

Computation on the D-wave is first a process of mapping the problem to the Ising structure, binary and quadratic, then embedding it into the available qubit lattice. On the D-wave the qubits are arranged according to a chimera graph, as illustrated in Fig 1. Each qubit couples to five or six others, except where there are defects due to faulty qubits. If the problem doesn’t embed directly, auxiliary qubits can be introduced to augment the available couplings, at a significant cost in qubits. Both mapping and embedding imply restrictions on the types of problems that can profitably be tackled with the D-wave processor. We will investigate these issues in the context of the QBoost algorithm. For a thorough recent study, and for more details on quantum annealing in the D-wave processor, see [22, 28].

## Boosting

Boosting is the tactic of building a strong classifier as an optimally weighted combination of weak classifiers, each of which may classify only moderately better than random guessing on its own. If the weak classifiers are linear in the input features, the boosted classifier carves out a piecewise-planar decision surface that is, if not to the same degree as that expressed by a neural network, effectively nonlinear. In 2008 Neven, Denchev, Rose, and Macready proposed a boosted classifier, christened QBoost, that could be trained on a D-wave processor [15]. Given *N* binary weak classifiers *c*_*i*_, *i* = 1…*N*, each of which classifies a data sample *t* according to *c*_*i*_(*t*) ∈ {−1, +1}, they sought a strong classifier of form

$$\begin{array}{c} C\left(t\right)=\text{sign}\left(\sum_{i=1}^{N}w_{i}c_{i}\left(t\right)\right). \end{array}$$ (3)

The authors achieved their best test results with binary weights, *w*_*i*_ ∈ {0, 1}, in which case the strong classifier is simply an optimal voting subset of weak classifiers. The natural cost function to mate with the D-wave architecture is a regulated quadratic loss. For a set *T* of training samples, with each element *t* having been assigned a training label *y*(*t*) ∈ {±1}, a training problem can be posed as follows:

$$\begin{array}{c} Find:\;min_{\left\{w_{i},\lambda \right\}}\left\{\sum_{t\in T}{\left(\sum_{i=1}^{N}w_{i}c_{i}\left(t\right)-y\left(t\right)\right)}^{2}+\lambda \sum_{i=1}^{N}w_{i}\right\}. \end{array}$$ (4)

The regularization term governed by the parameter *λ* is intended to improve generalization and speed in execution by keeping the final classifier compact. The normalization of the weak classifiers is then adjusted so as not to unduly penalize large positive margins from the decision hypersurface,

$$\begin{array}{c} c_{i}\left(t\right)\in \left\{-1/N,+1/N\right\}\;↔\;-1\leq \sum_{i=1}^{N}w_{i}c_{i}\left(t\right)\leq 1. \end{array}$$ (5)

The training problem thus formulated is one of quadratic unconstrained binary optimization (QUBO). In their initial tests of the algorithm, Neven et al. optimized the QUBO problem directly using classical heuristic solvers. Comparing with Adaboost, they found modest improvements in classification accuracy and significant improvment (of order 50%) in compactness of the boosted classifiers.

To convert the QUBO to Ising form, one makes the transformation *s*_*i*_ = 2*w*_*i*_ − 1. The new variables *s*_*i*_ take values *s*_*i*_ = ±1. Expanding the quadratic, the cost function becomes

$$\begin{array}{ll} & \sum_{i}\left(\lambda -2\sum_{t\in T}c_{i}\left(t\right)y\left(t\right)\right)w_{i}+\sum_{i,j}\left(\sum_{t\in T}c_{i}\left(t\right)c_{j}\left(t\right)\right)w_{i}w_{j}+const\\ \to & \sum_{i}\left(\frac{\lambda }{2}-\sum_{t\in T}c_{i}\left(t\right)y\left(t\right)+\frac{1}{2}\sum_{j,t\in T}c_{i}\left(t\right)c_{j}\left(t\right)\right)s_{i}+\frac{1}{2}\sum_{i>j}\left(\sum_{t\in T}c_{i}\left(t\right)c_{j}\left(t\right)\right)s_{i}s_{j}+const^{\prime }. \end{array}$$ (6)

In the latter equation, an extra factor of two in the quadratic term compensates for rewriting the sum to pass over all index pairs (*i*, *j*) once only. We can then identify the magnetic fields and couplings of the Ising frame Hamiltonian (Eq (2)),

$$h_{i}=-\frac{\lambda }{2}+\sum_{t\in T}c_{i}\left(t\right)y\left(t\right)-\frac{1}{2}\sum_{j,t\in T}c_{i}\left(t\right)c_{j}\left(t\right)$$ (7)

$$J_{ij}=-\frac{1}{2}\sum_{t\in T}c_{i}\left(t\right)c_{j}\left(t\right)$$ (8)

The constants dropped from Eq (6) do not affect the optimization. One can readily interpret how various terms influence the construction of the strong classifier. The contribution ∑_*t* ∈ *T*_ *c*_*i*_(*t*)*y*(*t*) to *h*_*i*_ describes how well the output *c*_*i*_(*t*) of a weak classifier correlates to the training labels *y*(*t*) over the training set *T*. If they correlate well, they give a strong positive contribution to the magnetic field, driving the spin to be positive. A positive spin indicates that the corresponding weight is equal to one: The weak classifier’s vote is tabulated in the final strong classifier. The coupling $J_{ij}=-\frac{1}{2}\sum_{t\in T}c_{i}\left(t\right)c_{j}\left(t\right)$ likewise describes the correlation of weak classifiers *c*_*i*_ and *c*_*j*_ over the training set. If the two weak classifiers correlate well, *J*_*ij*_ < 0. The spins *s*_*i*_ and *s*_*j*_ tend to opposite values, meaning one and not the other would be included in the final strong classifier. This is as it should be. To whatever extent they correlate, they supply redundant information on the data.

### Embedding into the chimera graph

The QBoost procedure results in a fully-connected Ising problem, with each *s*_*i*_ coupled to every other *s*_*j*_ by a (generically) non-zero *J*_*ij*_. To run on the D-wave processor the problem needs to be embedded into the chimera graph. The maximal degree of the chimera graph is six. The fully connected Ising problem on *N* spins constitutes a graph of degree *N* − 1. Nonetheless the latter can be embedded into the former by mapping each spin not to an individual qubit but to a connected subgraph of qubits, such that every subgraph (corresponding to an *s*_*i*_) is connected by at least one chimera graph edge to every other subgraph (corresponding to an *s*_*j*_) [31]. The graph edges bewteen subgraphs can be assigned the problem couplings *J*_*ij*_. Within a subgraph, internal graph edges can be assigned large, ferromagnetic couplings *J*_*F*_ to impose the condition that all qubits associated to a given spin align, encoding one and the same spin state.

This embedding comes at a high cost in qubits. Since each auxiliary qubit in a chimera subgraph couples to at most *d* other qubits, the subgraph size must scale with *N* to provide sufficient couplings to other subgraphs. As there are necessarily *N* subgraphs, the embedding overhead in qubits scales quadratically with the number of spins *N*. For the explicit examples studied recently in [32], *N* = 30 was the largest fully-connected problem embedable in a 512-qubit chimera graph. Much recent work [22, 24, 33–35] has gone into this and related embedding schemes, examining mappings of logical qubits to physical qubit subgraphs, optimal settings for the internal couplings *J*_*F*_, the distribution of problem couplings *J*_*ij*_ among graph edges, and more generally seeking problems that are less than fully connected and therefore more amenable to embedding in the chimera graph. Improving the connectivity of hardware graphs will be critical to broadening the scope of problems solvable on future quantum annealers.

In their 2009 demonstration of a QBoost classifier trained to detect cars in street scenes [17], Neven et al. embedded via a different approach. They mapped each Ising spin to a single qubit and discarded values *J*_*ij*_ that didn’t embed into the chimera graph. To this purpose they designed a greedy heuristic that assigns spins to qubits in succession, each spin to the qubit which will maximize the edge weight retained (the sum of the magnitudes of the embedded *J*_*ij*_) with respect to the previously embedded spins. Under this scheme they retained 11% of total edge weight on a 52-qubit embedding. (Only 52 qubits were functioning on the available D-wave processor, and they iterated training steps to grow a larger classifier.)

This strategy does not scale. Dropping too high a proportion of couplings leads to a scenario in which each spin variable can be optimized independent of the others. If, for a given spin *s*_*a*_, the magnetic field *h*_*a*_ is bigger than the sum of couplings to other spins *j* retained in the embedded lattice graph, i.e.,

$$\begin{array}{c} if\;|h_{a}|>\sum_{\{a,j\}\in E}\left|J_{aj}\right|, \end{array}$$ (9)

the value of *s*_*a*_ in the optimal solution is determined simply by the sign of *h*_*a*_. This can be seen by considering the total contribution to the energy due to spin *s*_*a*_, namely,

$$\begin{array}{c} E_{a}=-h_{a}s_{a}-\sum_{\{a,j\}\in E}J_{aj}s_{a}s_{j}. \end{array}$$ (10)

As in the preceeding equation, the sum here runs over the coupled spins *j*. If the spin *s*_*a*_ is anti-aligned with its magnetic field, the first term contributes −*h*_*a*_ *s*_*a*_ = +|*h*_*a*_| to the energy. Flipping the sign of *s*_*a*_ will decrease the contribution from that term by −2|*h*_*a*_|. At the same time, the second term is bounded,

$$\begin{array}{c} -\sum_{\{a,j\}\in E}|J_{aj}|\leq -\sum_{\{a,j\}\in E}J_{aj}s_{a}s_{j}\leq \sum_{\{a,j\}\in E}\left|J_{aj}\right|, \end{array}$$ (11)

and so flipping the sign of *s*_*a*_, regardless of the configuration of the other spins {*s*_*j*_}, imposes an energy cost of at most $+2\sum_{\{a,j\}\in E}\left|J_{aj}\right|$. When the condition (9) holds, flipping the spin leads to a net decerease of energy, and so the spin necessarily aligns with its magnetic field.

The consequences are two-fold. First, one can determine the optimal configuration of such spins simply by checking the signs of their magnetic fields. This is not a task that calls for a quantum computer. The implication for the classifier is the loss of fine balance that was to be achieved among all possible weak classifiers. We seek to retain only the minimal set of weak classifiers that captures the important features of the data, but weak classifiers whose spins satisfy condition (9) will be included or excluded irrespective of the inclusion of others.

Unfortunately, this scenario is to be expected as the total number *N* of input weak classifiers grows large. The base motivation for quantum computing is the hope that run times will scale better than for classical alternatives with the number of input variables. The effort only becomes justified on problems with thousands or tens of thousands of binary variables. At the same time, the number of connections between qubits (five or six in the case of the D-wave chimera graph) is likely to remain small, due to the challenge of building and controlling interactions between more than a few basic physical entities. For a problem with an initially fully connected graph, a simple one-variable-to-one-qubit embedding will discard thousands or tens of thousands of couplings against some some small finite number retained. Any computational problem that begins by imposing a quadratic loss function on a linear combination of binary variables, as in Eq (4), results in a fully connected graph. While some couplings may turn out to be zero, generically every spin couples to every other spin.

We can make these considerations more explicit by considering the scaling with *N* of the various terms in Ising Hamiltonian. Except in the case that the accuracy of weak classifiers is tuned close to 50%, the correlations ∑_*t* ∈ *T*_ *c*_*i*_(*t*)*y*(*t*) will be *O*(|*T*|/*N*), with |*T*| the size of the training set. For instance, in our implementation for tree cover classification, the average training error of the linear weak classifiers is 25%. A weak classifier with 25% training error would have

$$\begin{array}{c} \sum_{t\in T}c_{i}\left(t\right)y\left(t\right)=.25|T|\left(-1/N\right)+.75|T|\left(+1/N\right)=.5|T|/N. \end{array}$$ (12)

The *N* appears here through the normalization given in Eq (5). This level of training accuracy implies also that the weak classifiers are well correlated among themselves, with correlations that scale as

$$\begin{array}{c} \sum_{t\in T}c_{a}\left(t\right)c_{j}\left(t\right)∼O\left(\frac{\left|T\right|}{N^{2}}\right). \end{array}$$ (13)

Letting *k* be the maximum number of couplings between qubits in the graph G=(V,E), for large *N* we have the overall scaling rules:

$$|h_{a}|=\left|-\frac{\lambda }{2}+\sum_{t\in T}c_{i}\left(t\right)y\left(t\right)-\frac{1}{2}\sum_{j,t\in T}c_{i}\left(t\right)c_{j}\left(t\right)\right|∼O\left(\frac{\left|T\right|}{N}\right)\pm \frac{\lambda }{2}$$ (14)

$$\sum_{\left\{a,j\right\}\in E}\left|J_{aj}\right|=\sum_{\left\{a,j\right\}\in E}\left|-\frac{1}{2}\underset{t\in T}{\sum }c_{a}\left(t\right)c_{j}\left(t\right)\right|∼O\left(\frac{k\left|T\right|}{N^{2}}\right).$$ (15)

Since the regulator is fixed once for all spins and *k* is finite, a generic spin will satisfy the decoupling condition (9),

$$\begin{array}{c} |h_{a}|>\sum_{\{a,j\}\in E}\left|J_{aj}\right|, \end{array}$$

as *N* grows large.

**Fig 2:**
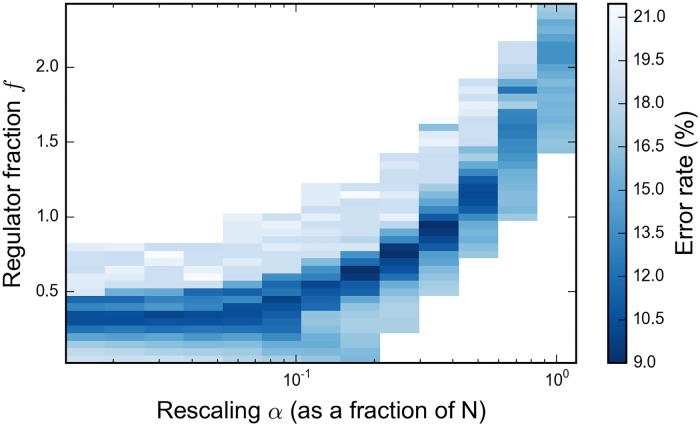
Validation error as a function of the coupling rescaling factor and regulator for the 108-qubit problem. The regulator is expressed in terms of a new parameter *f*: *λ* = 2*f*|*T*|/*N*. For each pair (*α*, *f*), the problem was optimized with 1000 calls to the D-wave processor and the classifier of minimal validation error recorded. The overall minimal error of 9%, in deepest blue, is attained for $\alpha \in \{\frac{N\sqrt{2}}{8},\frac{N}{4},\frac{N\sqrt{2}}{4}\}$.

We circumvented these difficulties, in the heuristic embedding scheme of Neven et al., by rescaling the retained couplings *J*_*ij*_ to compensate for those lost. The dynamics of Ising ferromagnets, in which long-range order appears in systems with only limited, local interactions, gave us reason to hope that a subset of five or six of *N* − 1 couplings, if appropriately rescaled, would be sufficient to maintain the characteristic balance sought between the weak classifiers. Absent a principled way to compute a rescaling on a spin-by-spin basis, we rescaled all couplings by a constant factor *α* which we treated as a new variational metaparameter. Intuitively, *α* should work out to be the ratio of lost to retained couplings, *α* ∼ *N*/5. (The current processor is constructed on an 1152-vertex chimera graph, with 55 currently inoperable qubits, making the average number of viable edges 5.6. Because the embedding heuristic maximizes the sum of magnitudes of retained couplings in preference to their number, the resulting embeddings are not maximally dense. Our embeddings typically retain an average of between four and five couplings per qubit.) A plot of validation error against the metaparameters of our 108-qubit problem, defined below in the section “Tree Cover Classification,” is shown in Fig 2. Stepping *α* by factors of $\sqrt{2}$ from *N*/64 to *N*, we find the solution of overall lowest validation error for $\alpha \in \{N\sqrt{2}/8,N/4,N\sqrt{2}/4\}$. This matches well with our expectations for *α* and situates the optimal classifier in the regime where the couplings and magnetic fields should have comparable, competing influence on the optimization. Moreover, we can see in the returned classifiers the increasing influence of the couplings with increasing *α*. When *α* is very small, the optimization is governed by the magnetic fields and the resulting classifiers consist predominantly of those weak classifiers which individually have lowest training error. To wit, the classifiers returned at the four smallest values of *α* share in common the twelve weak classifiers with the twelve lowest training errors; whereas, the optimal classifier realized for $\alpha \in \{N\sqrt{2}/8,N/4,N\sqrt{2}/4\}$ includes only two of those twelve; and the classifier at *α* = *N* includes one of the twelve. When we come to our results, we will explore these effects and the properties of the optimal classifier in more detail.

Incorporating the new rescaling factor, the energy function to be minimized across variables {*s*_*i*_, *α*, *λ*}, becomes, finally,

$$\begin{array}{l} H_{P}=-\sum_{i\in V}h_{i}s_{i}-\alpha \sum_{\left\{i,j\right\}\in E}J_{ij}s_{i}s_{j},\\ h_{i}=-\frac{\lambda }{2}+\sum_{t\in T}c_{i}\left(t\right)y\left(t\right)-\frac{1}{2}\sum_{j,t\in T}c_{i}\left(t\right)c_{j}\left(t\right)\\ J_{ij}=-\frac{1}{2}\sum_{t\in T}c_{i}\left(t\right)c_{j}\left(t\right). \end{array}$$ (16)

We will refer to the process of truncation and rescaling of the problem Hamiltonian as a renormalization, an abuse of a suggestive term from statistical physics. In thinking through this approach, it is worth remembering that we had already deviated from the most natural definition of the training problem at the point of imposing a quadratic objective function in place of the total number of misclassified training samples (*L*_2_ vs. *L*_0_ norm). We deviated again when we regularized the quadratic function. The choice of *L*_2_ over *L*_0_ norm is made habitually on grounds of computational tractability and justified ex post facto by the utility of the solutions that result. Likewise here, we look to the accuracy of the resulting classifiers to justify this reformulation of the original optimization problem. The most accurate classifier found for our 108-qubit problem using the renormalized Hamiltonian Eq (16) has a validation error rate of 9.00%. This compares to an error rate of 10.13% for the best solution found via simulated annealing on the original QBoost cost function. We have found that the final classifier can be improved if selected by validation in post-processing from among the outputs returned by the annealing process, and we do so as matter of course, although our results indicate that the effect diminishes for classifiers of larger cardinality.

Two final details of the implementation bear mention in the context of the embedding, for both of which we take cues from the original report on QBoost [15]. Along with the rescaling factor *α*, the regulator *λ* must be determined in training. Before submitting a problem for optimization, we specify the regulator in terms of a new parameter *f*,

$$\begin{array}{c} \frac{\lambda }{2}=\frac{f|T|}{N}. \end{array}$$ (17)

**Fig 3:**
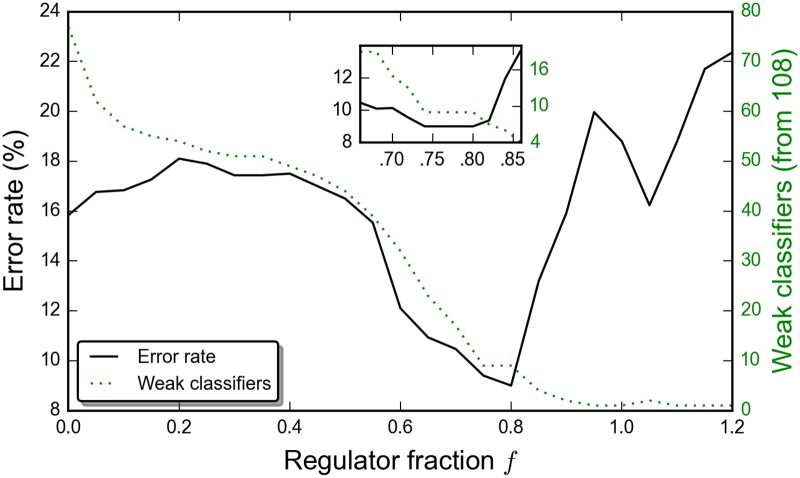
Minimum validation error and weak classifiers retained as a function of the regulator *λ* = 2*f*|*T*|/*N*. For each *f* the problem was optimized with 500 calls to the D-wave processor, and subsequently (inset), with 1000 calls in resolving the minimum, with *α* fixed at *N*/4.

Here, again, |*T*| is the number of training samples and *N* the number of input weak classifiers. The metaparameters (*α*, *f*) are chosen by acting the output strong classifiers on a 3000-sample validation set. (This step is coincident with the post-validation step mentioned in the previous paragraph.) Our practice has been to determine the fraction *f* initially by a coarse parameter scan and then to retest with finer step sizes around the minimum in *f*. The cardinality of weak classifiers in the strong classifier and its error rate depend strongly on *f*, as shown in Fig 3 with *α* fixed at *N*/4. The effect of the regulator for general *α* can be seen in Fig 2. Beyond enforcing compactness, the regulator evidently plays an important role in minimizing classifier training or validation error. With weak classifiers normalized so that *c*_*i*_(*t*) ∈ {−1/*N*, +1/*N*}, the quadratic loss,

$$\begin{array}{c} L={\left(\sum_{i=1}^{N}w_{i}c_{i}\left(t\right)-y\left(t\right)\right)}^{2}, \end{array}$$ (18)

favors margins (∑*w*_*i*_ *c*_*i*_) approaching values *y*(*t*) = ±1. It can work out that the average loss over the training set decreases as more weights are set to one, even as more samples are misclassified. This effect can clearly be seen in the optimization of the unregulated QBoost problem, in the “Results” section below. A finely-tuned regulator can neutralize this propensity to mostly non-zero weights. Alternatively, the tuning may be ameliorated and the problem given a more natural definition were the weak classifiers to be normalized such that $c_{i}\left(t\right)\in \left\{-1/\sqrt{N},+1/\sqrt{N}\right\}$. This would result in couplings *J*_*ij*_ suppressed by $1/\sqrt{N}$ relative to the magnetic fields *h*_*i*_ (cf. Eqs (14) and (15) where the factor is 1/*N*). This is the appropriate relative scaling for a fully-connected antiferromagnetic problem, such as we encounter prior to embedding.

Selection by validation entails significant processing overhead, and one would prefer to train the regulator online, with the weights. If it is to regulate all of the weights on equal footing, however, it must necessarily couple to all of them in the embedded problem. This would require a nontrivial but plausible investment of auxiliary qubits. It is only one parameter, and at four-bit depth it could be determined with necessary precision after a coarse scan. This seems to be worth exploring.

Similar considerations hold with respect to a training a bias shift for the decision hypersurface. The strong classifier can be improved by introducing a bias *B*, so that

$$\begin{array}{c} C\left(t\right)=\text{sign}\left(\sum_{i=1}^{N}w_{i}c_{i}\left(t\right)-B\right). \end{array}$$ (19)

We set *B* in post-processing as the average of the unbiased strong classifier on an additional 3000-sample class-balanced dataset.

## Tree cover classification

We have framed the problem of identifying tree cover in remote sensing imagery as a problem in machine learning. Given an ensemble of simple, and not necessarily very accurate, metrics on the image data that offer hypotheses as to whether pixels are covered or not covered by trees, the computer is to learn an optimal subensemble whose aggregated assignments identify tree cover more accurately than any of the metrics on its own. One of the advantages of this boosting approach, in principle, is that it can profitably be employed with any weak classifiers that perform better than random guessing. Insofar as the work reported on here is to function within a larger framework to delineate tree cover in 1-m-resolution NAIP aerial imagery, it inhereted a natural set of weak classifiers on the derived color and texture features employed therein.

In that framework [18], binary classification is performed by a fully-connected feed-forward neural network, namely, a multilayer perceptron. (An improved deep belief network for multiple-class cognition was studied in [36].) The inputs to the network are color and texture features extracted from eight-pixel-by-eight-pixel squares. These include standard statistical moments and Haralick features [37, 38] built on hue, saturation, intensity, and near infrared (NIR) bands, along with derived vegetation indices. The top 22 features, as ranked by a distribution separability criterion, are fed into the input layer of the network. There follow two hidden layers of ten neurons each and a single output neuron, which signals the probability that the land corresponding to the input image region is covered by trees. It is the functionality of this neural network, as abstracted from the larger processing pipeline developed in [18], that we are seeking to compliment and compare with the renormalized QBoost classifier.

We built weak classifiers as trained linear decision stumps on the inherited color and texture features. Explicitly, the stumps take the form

$$\begin{array}{cc} \left(x_{i}-b_{i}^{+}\right) & \geq 0\\ \left(-x_{i}-b_{i}^{-}\right) & \geq 0. \end{array}$$ (20)

Here *x*_*i*_ and *x*_*j*_ are the *i*th and *j*th components of the raw feature vector, and the *b*^±^ are trained thresholds. The training of each stump runs in time *O*(|*T*|log|*T*|), requiring a sort and two passes over the dataset of size |*T*|.

Training data were drawn from 537 of the 11,095 NAIP image tiles covering the state of California, roughly 5% of the whole, exhibiting dense tree cover, sparse tree cover, urban space, and barren lands. NAIP imagery is subject to stringent compliance guidelines and comes radiometrically corrected, which allowed us to assume a consistent calibration within the year-2012 dataset. We avoided clouds but admitted shadows as a source of error in the data. At eight-by-eight meters in size, a tree-labeled datum typically represents a contiguous grouping of trees, but may equally represent small trees or shrubs, given the lack of canopy height data for the bulk of our study region. Labelings were generated via an interactive segmentation tool based on a Random Walk algorithm [39], in which segments were seeded, labeled, and in some cases overdrawn by a user with expert domain knowledge. Within the protocol of [18], the training database was updated on the fly: For every hundred tree maps generated, ten tiles were selected at random and the interactive labeling tool applied to relabel misclassified examples, which were then incorporated into the training set with the correct labeling. This led to a training corpus weighted toward latitude 41°N, in the far-northern, densely tree-covered regions where class discrimination is most straightforward for human experts, but sampled from the entirety of the state. Further details on the development of the training data are given in [18].

For the tests reported on here, we extracted a total of 112 features from 30,000 labeled 8pixel × 8pixel squares, of which 24,000 were designated as training data points. Of these, 10,199 were positive class instances and 13,801 were negative class instances (tree covered or not, respectively). Two remaining 3000-sample sets were reserved for validation and bias determination. In limited testing with an additional 74,000 training samples drawn from the same 537 NAIP tiles, we found no improvement in validation and a slight degredation in performance on our test dataset.

Working with 112 features, one has a priori 224 linear decision stumps. Many of these perform no better than random guessing. These we discarded to save qubit resources. Ranked by training error, the eleven most accurate weak classifiers are stumps trained on various statistics of hue, with training error rates between 17.75% and 19.85%. The first several are given in Table 1. The effectiveness of hue in discriminating between trees and other types of land cover may reflect the arid conditions in California, with its extensive deserts and dry grasslands, when the data were captured. The next best weak classifier was the postive stump for the Atmospherically Resistant Vegetation Index (ARVI), with an error rate of 19.97%. These initial training steps provided an ensemble of 108 weak classifiers for input to the boosting algorithm for optimization on 108 qubits.

**Table 1:** Linear decision stumps with training error rates under 20%.

Underlying feature	Training error rate
Hue CCM^†^ entropy	.1775
Hue CCM 2nd Moment	.1788
Hue CCM energy	.1788
…	
[7 more derived from hue]	
…	
Hue CCM autocorrelation	.1985
Atmospherically Resistant Vegetation Index (ARVI)	.1997
mean (108 stumps)	.2545

^†^CCM stands for Color Co-ocurrance Matrix.

### Results

We focus on the 108-qubit problem problem because it clearly illustrates the mechanisms of the the algorithm, even though this particular instance exhibits fine-tuning effects. The best solution found misclassifies 270 samples from the 3000-sample validation set, an error rate of 9.00%. The errors are balanced between false positives and false negatives within half a percentage point. Since the 3000-sample validation set was used to select the classifier from among the solutions returned in annealing, we checked its performance on an additional 10,000-sample set, in no way used in the training but drawn from the same NAIP tiles as the training data. The performance on this set degrades slightly, to an error rate of 9.38%. The overall error rate is roughly half that of the best weak classifier alone, and further, the strong classifier is quite compact. Nine of 108 weak classifiers are retained. They are listed in Table 2.

**Table 2:** Linear decision stumps retained in solution to the 108-qubit problem.

Underlying feature	Training error rate
Hue CCM autocorrelation	.1985
Atmospherically Resistant Vegetation Index (ARVI)	.1997
Hue CCM sum of squares variance	.2085
NIR CCM sum entropy	.2094
Normalized Difference Vegetation Index (NDVI)	.2142
Simple Ratio (SR)	.2142
Saturation CCM homogeneity	.2196
Hue CCM contrast	.2251
Hue standard deviation	.2293

While all nine of the retained weak classifiers classify more accurately than the average weak classifier, only two, ARVI and the autocorrelation of the hue color co-occurance matrix (CCM), figure in the list of top-ranked weak classifiers in Table 1. The most accurate weak classifiers on this dataset are all derived from hue, and therefore they are fairly redundant discriminants of tree cover. The nine weak classifiers selected, on the other hand, are built on hue, saturation, and near-infrared bands, along with three vegetation indices. This is precisely the desired effect of boosting. The goal is to have the computer select a minimal subset of weak classifiers that together capture the diverse important dependencies in the data. A simple measure of the similarity of weak classifiers is their correlation on the training dataset,

$$\begin{array}{c} Corr\left(i,j\right)=\frac{N^{2}}{\left|T\right|}\sum_{t\in T}c_{i}\left(t\right)c_{j}\left(t\right), \end{array}$$ (21)

normalized so that *Corr*(*i*, *i*) = 1. Among the nine most accurate weak classifiers the median correlation is.86, while among the nine classifiers selected in the boosted solution, the median correlation is .44. A median of .44 is not exceptionally low, rather, it is precisely the median correlation across all pairs drawn from the 108 weak classifiers.

To compare the performance of the D-wave processor with simulated annealing, we fixed the embedding and fixed the regulator fraction (cf. Eq (17)) corresponding to the minimum validation error in Fig 3. The problems submitted to each optimization method were thus identical, the only variability arising from randomness within the optimization methods themselves. For simulated annealing, we instantiated a random initial configuration of spins and used a linear annealing schedule with up to two thousand temperature steps, *N* spin flips per step. The starting temperature was chosen as the maximum change in energy associated with any single spin flip from the initial configuration. The annealing stopped when the two thousand steps were exhausted or after there was no change in energy at three distinct temperatures. In settling on this program we took our cues from [40]. We did not endeavor to replicate the nuanced experiments performed elsewhere [11–13] comparing processing times for simulated annealing against the those for the D-wave processor, rather wanting to compare the distributions of returned results. It is probably of interest to note, however, that all anneals on the D-wave machine were performed in the default time of 30*μ*s, not including cooling, initialization, and read out times. Because of queuing for the machine and extensive classical processing pre- and post-anneal, the wall times for the tests reported on here were on the order of hours.

**Fig 4:**
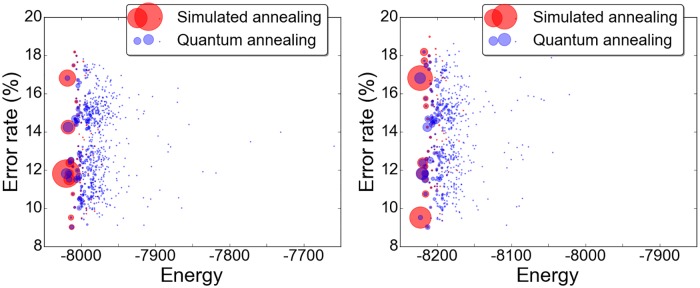
Quantum vs. simulated annealing on our 108-qubit problem, with *α* = *N*/4. Plots show two thousand anneals with each method for each of two regulators (*f* = .76, left, and *f* = .77, right). The area of each marker is proportional to the number of times the given solution occurs.

Scatter plots of results from two thousand anneals with each method are shown in Fig 4, repeated with slight adjustment to the regulator to indicate the shifting quality of the solutions. The stochastic nature of both optimization methods is clearly visible in the results. In the case of the D-wave processor, randomness enters both through the finite precision with which magnetic fields and couplings can be applied to the qubits as well as thermal noise in the device. There is significantly more variance in the results returned by the quantum annealer, although both methods find the same compliment of the few lowest energy states. The two methods return the solution of lowest validation error at comparable rates.

**Fig 5:**
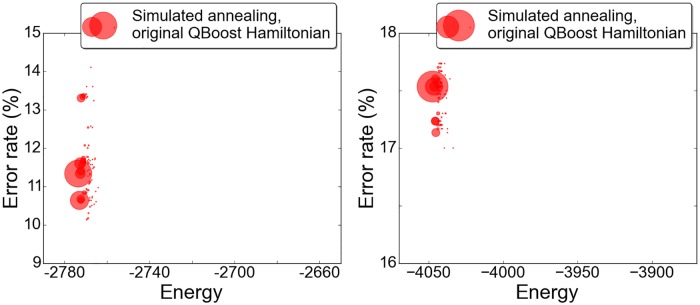
Results for the 108-variable problem using the original QBoost objective function. At left, with selected regulator fraction *f* = .44, the solutions include 26 to 29 weak classifiers. At right, for comparison, the solutions with *f* = 0 include 93 to 96 weak classifiers and demonstrate (by its absence) the essential role played by the regulator in minimizing classifier error. Note the differences in vertical scale, here and with respect to Fig 4. The horizontal axes are proportionally scaled.

By simulated annealing, it is also possible to optimize the original QBoost cost function without embedding into the chimera graph, and without having to prune and rescale couplings as a consequence. For this problem the regulator fraction selected was *f* = .44, which admits solutions in the range of 26-29 weak classifiers. Results from two thousand anneals are shown in Fig 5. The solution with minimal error on the validation set included 28 weak classifiers and yielded an error rate of 10.12%. Looking for the best known solution, we also artificially forced the regulator into the range that would allow solutions with nine weak classifiers. Across many thousands of anneals, no solution was returned with validation error less than 14%. For comparison, we also include results for the QBoost objective with zero regulator in Fig 5.

One striking feature of the scatter plots is the wide range of validation errors among solutions returned at energies differing by only a fraction of a percent. The range of validation errors is broader for the renormalized problem, but the issue exists also for the original. It may simply be an artifact of the small number of weak classifiers retained in this case. For instance, the two lowest-energy solutions at right in Fig 4, with validation errors 16.80% and 9.50%, respectively, share an identical compliment of seven weak classifiers, to which the former but not the latter adds an eighth. One vote added to seven can significantly impact the result. In the tests that have returned larger numbers of weak classifiers (cf. Fig 5, right), the variance in validation errors among the low energy states is much reduced. Still, it must be noted that the *L*_2_ norm is not always a reliable proxy for *L*_0_ norm, and selection of an optimal classifier in post-validation is recommended by the distribution of results. This is not an entirely satisfactory state of affairs. In effect, the quantum or simulated annealing is serving to sample the low-lying energy states of the problem Hamiltonian rather than to find a unique minimal energy state. At least at the optimal value of the coupling rescaling *α*, the solution of lowest validation error is near enough to minimum energy that it is returned reliably among the solutions: The solution with 9% validation error appears some twenty to thirty times per two thousand anneals (Fig 4). As actualized in this instance, the algorithm relies on the stochastic nature of the quantum and simulated annealing solvers to find the best possible classifier.

### Feature expansion

By expanding the feature set to include quadratic products of the input features, one can add a measure of nonlinearity to the classifier. This possibility was explored already in the original work on QBoost [15]. For pairs of input features *x*_*i*_, *x*_*j*_, the quadratic decision stumps are defined by

$$\begin{array}{cc} \left(x_{i}x_{j}-b_{ij}^{+}\right) & \geq 0\\ \left(-x_{i}x_{j}-b_{ij}^{-}\right) & \geq 0, \end{array}$$ (22)

for trained thresholds $b_{ij}^{\pm }$. Many of these quadratic stumps are quite accurate in and of themselves. The product of ARVI with a mid-frequency discrete cosine transform (DCT) returns error rates of 9.8% in training and 10.9% on the validation set. The product of ARVI with the standard deviation of the NIR band yields training and validation errors of 10.6% and 10.9%. Ten of the twelve most accurate quadratic stumps pair a vegetation index with a Haralick feature or statistical moment. It is well known that using vegetation indices in combination with another feature on the data can improve classification accuracy significantly over the vegetation index alone. The quadratic stumps used here are a particularly simple execution of this idea. Training a stump requires a sort and two passes over the training data and can be executed in some ten or tens of lines of code. Where speed and simplicity are a priority, the quadratic stumps may serve as creditable stand-alone classifiers.

Inputting 112 features to Eqs (20) and (22), one has a priori

$$2\left(112+\left(\begin{array}{c} 112\\ 2 \end{array}\right)\right)=12656$$

linear plus quadratic decision stumps. In order to train on a D-wave processor with 1097 functioning qubits, one needs either to train iteratively or to reduce the number of input features. We pursued the latter option. We selected a combination of thirty features whose quadratic stumps yielded the lowest training error rates, the highest training error rates (after discarding random guessers), and pairs with lowest mutual correlation in the sense of Eq (21), hoping by this minimal artistry to begin with a set of weak classifiers that express a wide range of opinions on the data. Along these lines, Pudenz and Lidar [41] formalize criteria under which a strong classifier with bounded error may be constructed from pairs of weak classifiers that disagree in their classification on all but small subsets of feature space. Minimal correlation between weak classifiers, in the sense we are using it, is a rough practical proxy for their more formal criteria. The thirty features thus chosen include a range of derivatives of hue, saturation, intensity, and NIR bands, along with ARVI, the Normalized Difference Vegetation Index (NDVI), Simple Ratio (SR), and Enhanced Vegetation Index (EVI). With random guessers discarded, the linear and quadratic stumps on these features yield a compliment of 508 weak classifiers.

**Fig 6:**
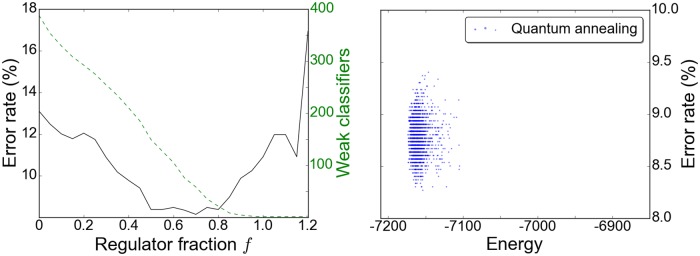
Results on the 508-quibt problem, with *α* = *N*/5. Left: A coarse scan for the regulator fraction *f*. Right: Output from two thousand anneals with *f* = .70.

The optimal solution found to the 508-qubit problem has rescaling factor *α* = *N*/5, regulator fraction *f* = .70, and retains 52 of 508 input weak classifiers. It yields an error on the 3,000-sample validation set of 8.27%, improving fractionally to 8.25% on the longer, independent 10,000-sample set. With the larger set of weak classifiers, the results are much less sensitive to small variations in the metaparameters and in the weak classifiers included in the boosted classifier, as can be seen by comparing Fig 6 with the corresponding output for the 108-qubit problem (Figs 3 and 4). Though we continued to extract the best solution by post-validation, the lowest-energy metric now yields near-lowest validation errors. In this instance the lowest energy solution has a validation error of 8.80%. The modest improvement of these solutions over the individual quadratic decision stumps and the boosted linear stumps suggests a limit to the gains achievable with piecewise, low-polynomial-degree nonlinearity.

**Fig 7:**
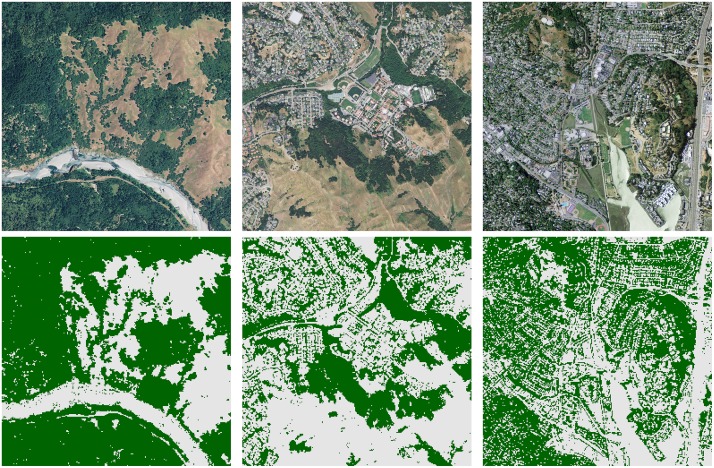
Classification of tree cover by boosted linear-plus-quadratic stumps, from the 508-qubit problem. Left: A region of broken tree cover outside the town of Blocksburg, CA. Middle: Saint Mary’s College of California. Right: The city of Mill Valley, CA.

Applying the optimal solution to an area of broken tree cover near the town of Blocksburg in northwest California, and then to the surburban and ranch lands around Saint Mary’s College, yields the output classifications shown in Fig 7, left and middle. In the second scene, coarse graining due to feature extraction on eight-by-eight pixel squares causes the tree cover to be overestimated in regions where trees are interspersed among buildings. The classifier is largely successful in discriminating between the green lawns and playing fields of the college and the textured tree cover of the hillsides. The third panel shows a densely built area in the San Francisco Bay Area city of Mill Valley.

**Fig 8:**
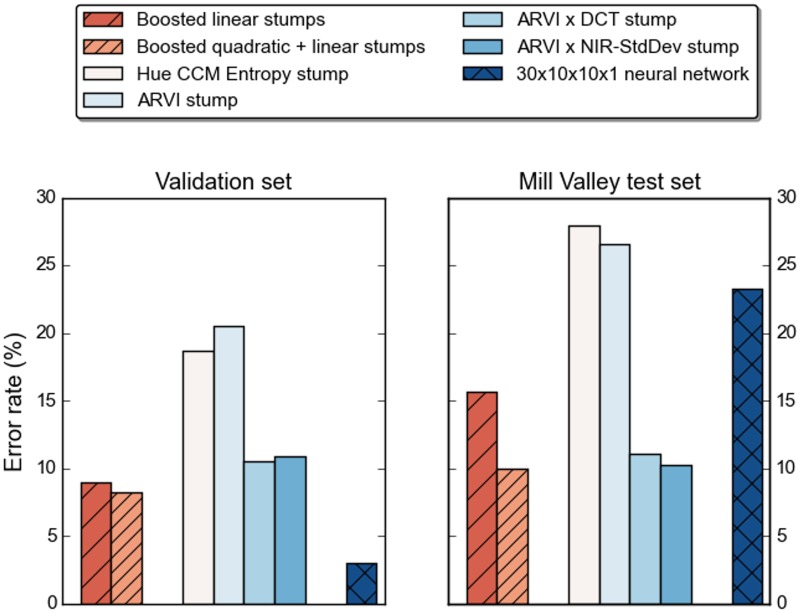
Error rates for the boosted classifiers vs. individual weak classifiers and a 30x10x10x1 neural network.

We selected the NAIP tile containing the Mill Valley scene to develop a dataset for additional testing, seeking the challenge of its highly spatially mixed land-cover classes. Further, densely built areas constitute a relatively small proportion of total land area in the state, and thus, of the training data. Of 24,610 labeled data points from the Mill Valley tile, 5,176 were identified as tree cover and 19,434 as other forms of land cover. We benchmarked performance of the two boosted classifiers (solutions to the 108- and 508-qubit problems) against the two most accurate linear decision stumps, the two most accurate quadratic decision stumps, and a neural network. The neural network was a fully-connected multilayer perceptron taking as input the same thirty features used to generate the 508-qubit problem, with two hidden layers of ten neurons each. The results appear in Fig 8. The gains from boosting over individual weak classifiers are clear in validation, where at the same time the neural network far and away outperforms. On the Mill Valley scene, the results for the neural network demonstrate a distinct tradeoff between fit to the training data and generalization to this test data. The boosted classifier built on linear-plus-quadratic stumps, and to a lesser extent the individual quadratic stumps, perform moderately well on both datasets.

Vegetation indices have long served as the operative standard for detecting photosynthetic activity in remote sensing imagery. Our platform includes four indices (NDVI, SR, EVI, ARVI) as base features, made weak classifiers with trained thresholds. The boosted classifiers handily outperform these indices. On the validation set, the linear-plus-quadratic boosted classifier has an error rate of 8.27%, against 20.5% for ARVI. On the Mill Valley test set its error rate is 10.00% vs. 26.56% for ARVI. The comparisons are less favorable for the other vegetation indices, with the exception of a test set error of 25.83% for EVI. At the same time, the data suggest that one can capture much of this advantage by combining ARVI with one other feature. To wit, in a quadratic stump with a discrete cosine transform its validation and test set errors are 10.5% and 11.13%, or with NIR standard deviation, 10.9% and 10.25%. As we noted above, these quadratic stumps are nearly as simple to deploy as the vegetation indices themselves and on this evidence merit further testing.

## Discussion and conclusions

This work began as an attempt to envision the possibilities and challenges that may be encountered in future applications of quantum annealing to environmental remote sensing. We set for ourselves a case study, to leverage available quantum annealing hardware manufactured by D-wave Systems to identify tree cover in very high resolution aerial imagery. The constraints dictated by the hardware are significant. To formulate a problem for optimization on the current D-wave processor, one must consider that:

1. The programmable variables are binary and finite in number.
2. The programmable objective functions are quadratic in the binary variables.
3. The number of non-zero quadratic coefficients for any variable is limited to six. These coefficients, along with the variables themselves, must be mapped (embedded) into the edges and vertices of a degree-six chimera graph.

While it is possible to encode floating point numbers in binary digits (point 1) and, using auxiliary variables, to reduce higher-order polynomials to quadratic (point 2), these workarounds exacerbate the embedding problem (point 3). These issues stand quite apart from questions surrounding the performance of the hardware, and in particular the extent to which quantum coherence is maintained among qubits. As hardware matures, we may very well see more robust quantum coherence among larger sets of qubits. Unless the graph connectivity increases as well, large and largely connected problem instances, as are generated by the broad class of quadratic training objectives of the form given in Eq (4), will continue to be difficult to embed and therefore optimize directly in quantum annealing. It may indeed be some time before the community identifies the class of problems which best leverage the unique capabilities of a quantum annealing processor.

Nonetheless, by truncating and rescaling the couplings in a regulated quadatic training objective, we were able to train on the D-wave processor a binary tree-cover classifier. We offered intuition for the modifications to the objective function, but in the end, we had to rely for justification on the efficacy of the results. The argument for the approach would be stronger if the lowest energy were a more reliable predictor of lowest validation error, thus obviating the need to select among solutions by post-validation; but then, the same critique can be leveled at the original regulated quadratic QBoost objective. It stands to reason that a non-convex loss function more nearly approximating 0-1 loss would help. In seeking a loss function robust to label noise, [42] considered a doubly-truncated quadratic loss which can be approximated, in upper bound, by a family of quadratic functions. This truncated quadratic loss is therefore trainable on the D-wave processor, with an additional metaparameter to complicate the embedding, and might serve as a more suitable starting point. If the training penalty tapers off with distance from the decision hypersurface, this would also relieve the problem of fine-tuning the regulator, noted above. Another immediate improvement to the training scheme would be to employ auxiliary qubits to embed and train online important metaparameters, such as the regulator and coupling rescaling factor.

For our prototype 108-qubit problem, the trained classifier incorporates an array of metrics based on hue, saturation, and NIR bands, along with vegetation indices, which together discriminate tree cover with accuracies of 91% in validation and 84% on the Mill Valley test set. A validation error rate of 9% cuts by half the error rate from the best of the weak classifiers on their own. The boosted classifier is compact, relatively robust in generalization, and fast in execution: After feature extraction, a sample datum can be classified by tabulating nine less than / greater than comparisons. By feature expansion, the accuracy can be improved to 92% in validation and 90% on the Mill Valley test set. The performance of the classifier likely could be improved further by incorporating a broader set of weak classifiers, in hopes of better capturing the multivalent dependencies of the data, and by increasing the nonlinearity available to the system as expressed in the weak classifiers. The piecewise-polynomial nonlinearity available to boosted decision stumps will never achieve the complex transformations of the input data space that are possible in a deep neural network, and a multilayer perceptron already fits our training data better than does the boosted classifier. As deep learning frameworks grow in complexity, boosting may prove useful to preselect features to input to such networks. [43]

In sum, we were able with some effort to construct a viable classifier of tree cover, despite the restrictions posed by the hardware architecture. Whether this framework proves compelling in the long run will depend on the maturation of quantum annealing hardware, the gains to be found in larger ensembles of input metrics, and the relative challenge of training competing frameworks at similar scale.
